# Prevalence and pattern of HIV-related malnutrition among women in sub-Saharan Africa: a meta-analysis of demographic health surveys

**DOI:** 10.1186/1471-2458-8-226

**Published:** 2008-07-02

**Authors:** Olalekan A Uthman

**Affiliations:** 1 Center for Evidence-Based Global Health, Save the Youth Initiative, Nigeria

## Abstract

**Background:**

The world's highest HIV infection rates are found in Sub-Saharan Africa (SSA), where adult prevalence in most countries exceeds 25%. Food shortages and malnutrition have combined with HIV/AIDS to bring some countries to the brink of crisis. The aim of this study was to describe prevalence of malnutrition among HIV-infected women and variations across socioeconomic status using data from 11 countries in SSA.

**Methods:**

This study uses meta-analytic procedures to synthesize the results of most recent data sets available from Demographic and Health Surveys of 11 countries in SSA. Pooled prevalence estimates and 95% confidence intervals were calculated using random-and fixed-effects models. Subgroup and leave-one-country-out sensitivity analyses were also carried out.

**Results:**

Pooling the prevalence estimates of HIV-related malnutrition yielded an overall prevalence of 10.3% (95% CI 7.4% to 14.1%) with no statistically significant heterogeneity (*I*^2 ^= 0.0%, p = .903). The prevalence estimates decreased with increasing wealth index and education attainment. The pooled prevalence of HIV-related malnutrition was higher among women residing in rural areas than among women residing in urban areas; and lower among women that were professionally employed than unemployed or women in agricultural or manual work.

**Conclusion:**

Prevalence of HIV-related malnutrition among women varies by wealth status, education attainment, occupation, and type of residence (rural/urban). The observed socioeconomic disparities can help provide more information about population subgroups in particular need and high risk groups, which may in turn lead to the development and implementation of more effective intervention programs.

## Background

An estimated 33.2 million [30.6 million – 36.1 million] people worldwide were living with HIV in 2007 [1]; 2.5 million [1.8 million – 4.1 million] became newly infected with HIV; and 2.1 million [1.9 million – 2.4 million] lost their lives to AIDS [1]. Sub-Saharan Africa (SSA) continues to be the region most affected by the AIDS epidemic; nearly 22.5 million [20.9 million – 24.3 million] adults and children had HIV/AIDS in 2007 [1]. More than two out of three (68%) adults and nearly 90% of children infected with HIV live in this region, and more than three in four (76%) AIDS deaths in 2007 occurred in SSA [1]. It is increasingly clear from the research that young women in sub-Saharan Africa are at particularly high risk of HIV infection. It is also important to note that women bear the greatest burden of frequent high-risk pregnancies, raising large families. Together, these conditions have had devastating consequences for the health and well-being not only of African women but also their families. In sub-Saharan Africa, almost 61% of adults living with HIV in 2007 were women [1].

Malnutrition rates are increasing in the African region [2]. Food shortages and malnutrition have combined with HIV/AIDS to bring some countries to the brink of crisis [2]. Furthermore, food is often identified as the most immediate and critical need by people living with HIV/AIDS and others affected by the pandemic [2]. Describe since the outset of the AIDS pandemic [3], malnutrition is frequent and a marker for poor prognosis among HIV-infected subjects [4-6]. HIV-related malnutrition has several causes [7], including but not limited to a decrease in food intake, the effects of opportunistic infections, metabolic inefficiencies due to cytokine activity and diarrhea. Malnutrition itself can induce immuno-depression [8] and worsen HIV-related immuno-depression [9].

African governments are currently grappling with a range of programme and policy challenges related to food, nutrition and HIV/AIDS [2]. Hunger and Malnutrition are major causes of the deprivation and suffering targeted by all other millennium development goals (MDGs) [10]. Logic suggests, and ample evidence confirms, that broader strategy for meeting MDG 1 (hunger and malnutrition) put forward at United Nations Economic and Social Council (ECOSOC) will also serve to accelerate progress towards the other MDGs [10]. One vital element in improving this situation is the need for a comprehensive and relevant evidence base that would equip SSA countries to take informed actions. Few studies [3,11-13] have been dedicated to the prevalence of malnutrition among Africa adults, although it is a problem increasingly faced by practitioners in places with a high prevalence of HIV infection. These studies consisted of autopsy series of HIV-seropositive subjects [13]; or specifically focused on cachectic HIV-seropositive patients [3] or tuberculosis patients [11,12], but none evaluated the burden of malnutrition among general populace. In addition, there are currently no pooled data available stratifying prevalence based on wealth status, occupation, education attainment, or type of residence (urban/rural), all which can affects risk of malnutrition among HIV-positive subjects. Without objective information about the current patterns of malnutrition among HIV-infected people, it is difficult to plan substantial public health programs that could prevent and improvement nutrition in HIV infected people. The aim of this study was describe prevalence of malnutrition among HIV-infected women and variations across socioeconomic status.

## Methods

### Data and selected countries

This study uses the most recent data sets available as of October 2007 from the Demographic and Health Surveys (DHS) of the following 11 countries: Burkina Faso, Cameroon, Ethiopia, Ghana, Guinea, Kenya, Lesotho, Malawi, Rwanda, Senegal, and Zimbabwe. The data sets were chosen based on availability of women anthropometric measures and HIV test results to allow comparative analysis. The DHS data sets are from nationally representative surveys of households with at least one woman of reproductive age, usually between 15 to 49 years. The data were collected by various in-country research and statistical agencies with technical assistance from ORC Macro International, Inc (Calverton, Maryland, USA). All eligible women in each selected household were surveyed. The surveys were based on two-stage sample designs. In the first stage, enumeration units or "clusters" were selected from larger regional units within countries. Next households were randomly selected within clusters.

### Variables

#### Main outcome

For this study, prevalence of HIV-related malnutrition among women was defined as proportion of HIV seropositive women with body mass index (BMI) less than 18.5 (according to World Health Organization cut-off for underweight [14]). All eligible women interviewed were also asked to voluntarily test for HIV. Women were provided with a voucher that entitles them to free counselling and testing at a nearby facility to ensure that they are in a position to know their HIV status if they want to [15]. HIV testing was done using a dried blood spot. The standard testing algorithm includes using two different HIV antibody Enzyme-Linked ImmunoSorbent Assays, ELISAs (E1 and E2). All discordant samples that were positive on the first test and negative on the second test (E1+ and E2-) were subjected to a second round of testing using both the ELISAs. The discordant samples from the second round of testing were classified as "indeterminate". The "indeterminate" samples were then subjected to a third confirmatory test using the Western-Blot. The Western-Blot result was considered final for the indeterminate samples. These steps were repeated for 5–10% of randomly selected samples that tested negative on the first test [16]. Body mass index (BMI), was calculated as weight in kilograms divided by height in meters squared (*kg*/*m*^2^). Weight was measured by using a solar-powered scale with an accuracy of ± 100 g, and height was measured with an adjustable wooden measuring board that is designed to provide accurate measurements (to the nearest 0.1 cm) in the context of a developing-country field situation [17].

#### Socioeconomic factors

The study considered four measures of socioeconomic position: wealth index, education, occupation, and place of residence. Wealth status is measured in the DHS surveys in terms of assets, rather than income. Ownership of consumer items, such as a radio or car, as well as characteristics of the dwelling, such as floor or roof type, toilet facilities, and water source, are items that measure the concept of poverty in these settings; this concept has been used by the World Bank to allocate households and thus their members into poverty quintiles, using principal components analysis (PCA) [18-22]. The weighted scores were divided into quintiles for the analytic models. The level of education attained was defined as never been to school, primary, and secondary or higher education. Women's current occupation was defined as being currently engaged in white collar work (e.g., professional and managerial positions, clerical or sales, or generally employed in the service sector), manual or agricultural work (including paid household or domestic work), and not currently participating in the labour force (including those not seeking work, such as homemakers). Place of residence was defined as rural or urban as they are defined for administrative purpose for each country.

### Ethical consideration

This study is based on an analysis of existing survey data with all identifier information removed. The survey was approved by the Ethics Committee of the ORC Macro at Calverton in the USA and by the National Ethics Committees of the 11 countries. All study participants gave informed consent before participation and all information was collected confidentially. The HIV testing protocol does not allow individual test results to be linked to a specific respondent, to safeguard the confidentiality of the information. Eligible women were informed about the procedures, the confidentiality of the result, and the fact that test results will not be made available to them.

### Meta-analysis

Apparent prevalence estimates were computed using number of HIV positive women with BMI <18.5 and the total number of HIV positive women reported in each country. Because a normal distribution is mandatory for the pooling of data, *logit *transformation was applied as outlined by Lipsey et al [23] and weighted by inverse variance of *logit *transformed prevalence. Two techniques were used to calculate the pooled prevalence estimates: the Mantel-Haenszel method [24] assuming a fixed-effects model and the DerSimonian-Laird method [25] assuming a random-effects model.

The *logit *effect size for prevalence, its standard error, and the inverse variance weight are:

(1) $${\text{ES}}_{l}={\text{log}}_{\text{e}}\left[\frac{p}{1-p}\right]$$

(2) $${\text{SE}}_{l}=\sqrt{\frac{1}{np}+\frac{1}{n(1-p)}}$$

(3) $$w_{l}=\frac{1}{SE_{l}^{2}}=np(1-p)$$

where *p *is the proportion of women with HIV-related malnutrition and *n *is the total number of women that are HIV seropositive in the sample. The final pooled *logit *results and 95% confidence intervals (CIs) were back-transformed to proportion for ease of interpretation using:

(4) $$p=\frac{e^{\text{logit}}}{e^{\text{logit}}+1}$$

To evaluate whether the results of the studies were homogenous, the Cochran's Q test was used [26]. The quantity *I*^2 ^that describes the percentage of variation across studies that are heterogeneity rather than chance was calculated [27,28]. Subgroup analyses were performed according to participants' socioeconomic characteristics: Wealth index, education attainment, present occupation, and type of residence. To evaluate the stability of the results and to test whether one country had an undue influence on the meta-analysis, leave-one-country-out sensitivity analysis was performed [29]. The scope of this analysis was to evaluate the influence of individual countries, by estimating prevalence of HIV-related malnutrition in the absence of each country. All tests were two tailed. For all tests, a probability level less than .05 was considered significant. Stata 10 (Stata Corporation, College Station TX) software was used for the statistical analyses.

## Results

### Description of included countries

This study uses data from 11 Demographic and Health Surveys (DHS) conducted between 2003 and 2006 in sub-Saharan Africa (SSA). The countries, years of data collection, and sample sizes are listed in Table 1. Table 1 also shows the proportion of women (both sero-negative and sero-positive) classified as underweight, normal weight, overweight, and obese for countries included in this study. Table 2 illustrates the demographic and economic diversity of the selected countries. Regarding levels of urbanization, the percentage of urban population differs significantly among the selected countries. It varies from 16% – 18% in Ethiopia, Malawi, and Burkina Faso, to more than 40% in Senegal, Ghana, and Cameroon. As for gross domestic product (GDP) per capita, Cameroon, Lesotho, and Senegal emerge as the most affluent countries with values higher than US$ 600, whilst by contrast Ethiopia and Malawi were the most deprived (less than US$ 200). The fastest growths were recorded in Rwanda (5%), Burkina Faso (3%), and Ethiopia (3%). By contrast, Lesotho (1%) and Zimbabwe (1%) witness the slowest growth rates of their urban population. The most marked reductions in GDP per capita from 1990 to 2004 were in Zimbabwe (-2%) and Kenya (-1%), whereas improvements were recorded in Lesotho (5%) and Ghana (2%) to a lesser degree in Cameroon (1%). The selected countries also displayed marked socioeconomic diversities in term of per capital health expenditure, adult literacy, and per capita food production (data not shown).

**Table 1:** Description of data sets, study sample size, proportion of women (both seropositive and seronegative) classified as underweight, normal, overweight, and obese for countries included in the study

			***BMI classification (all sample) (m^***2***^/kg)***			
			
**Region/Country**	**Year of survey**	**Sample size**	**Underweight (<18.5)**	**Normal (18.5–24.9)**	**Overweight (25.0 – 29.9)**	**Obese (≥ 30)**
**Central Africa**						
Rwanda	2005	11, 321	9.9	78.2	11.0	1.0
Cameroon	2004	10. 656	6.3	65.2	20.6	7.9
**Eastern Africa**						
Kenya	2003	8, 195	11.9	63.8	17.4	6.8
Ethiopia	2005	14, 070	26.6	67.0	5.2	1.2
**Southern Africa**						
Lesotho	2004	7, 095	5.5	53.9	25.5	15.1
Malawi	2004	11, 698	9.2	77.5	10.8	2.5
Zimbabwe	2006	8,907	9.1	66.1	17.9	6.9
**Western Africa**						
Burkina Faso	2003	12, 477	21.6	69.8	6.4	2.2
Ghana	2003	5, 691	9.8	67.2	15.8	7.3
Guinea	2005	7, 954	13.7	73.1	10.7	2.5
Senegal	2005	14, 602	18.1	61.6	14.0	6.2

BMI-Body mass index

**Table 2:** Comparative demographic and social indicators for selected 11 countries in Sub-Saharan Africa

	***Population***			***GDP per capital***	
	
**Region/Country**	**Total (1000)**	**% urban**	**Annual growth rate (%)**	**US$**	**% annual variation**
**Central Africa**					
Rwanda	9, 038	19	5.2	208	-0.1
Cameroon	16, 322	55	2.1	421	+0.5
**Eastern Africa**					
Kenya	34, 256	21	2.3	481	-0.6
Ethiopia	77, 431	16	2.6	114	+1.5
**Southern Africa**					
Lesotho	1,795	19	0.6	730	+4.5
Malawi	12, 884	17	2.5	149	+0.9
Zimbabwe	13, 010	36	1.0	363	-1.9
**Western Africa**					
Burkina Faso	13, 228	18	3.0	376	+1.3
Ghana	22, 113	48	2.2	409	+1.9
Guinea	9, 402	33	2.3	421	+1.0
Senegal	11, 658	42	2.5	683	+0.9

### Meta-analysis

**Figure 1:**
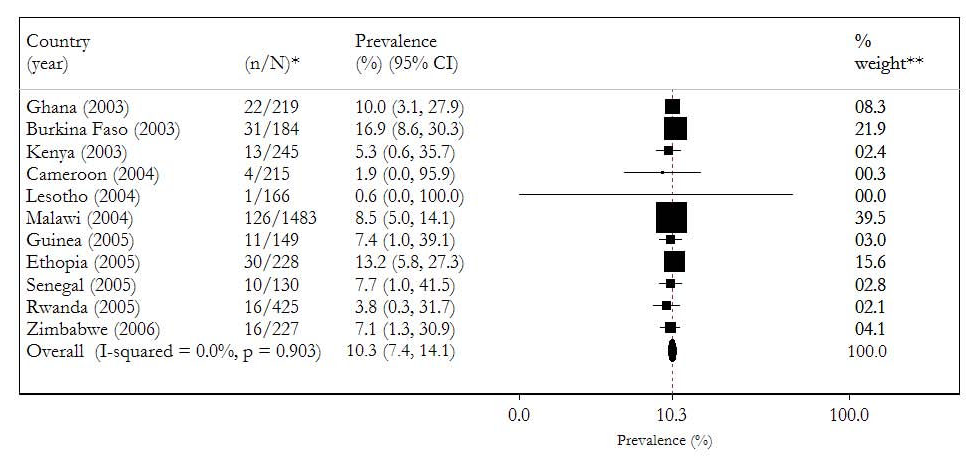
**Forest plot of meta-analysis of the prevalence estimates from 11 studies**. *HIV-related, proportion of HIV-seropositive women (N) that are underweight (n). **Pooled effect estimate is the same for both random- and fixed-effects model. The vertical line represents the prevalence estimates of the pooled result. ***Inverse variance weight.

The prevalence of HIV-related malnutrition among women (HIV-positive women with low body weight) varied widely, from 0.6% in Lesotho to 16.9% in Burkina Faso (crude unweighted mean 7.7%). Figure 1 graph the prevalence estimates and 95% confidence intervals (CIs) from the individual countries and pooled result. Meta-analysis of all 11 countries yielded an overall pooled prevalence of 10.3% (95% CI; 7.4% to 14.1%) with no statistically significant heterogeneity (Cochran's *Q *test = *Q *= 4.82 on 10 degree of freedom, P = .903, I^2 ^= 0%).

### Sensitivity analyses

**Figure 2:**
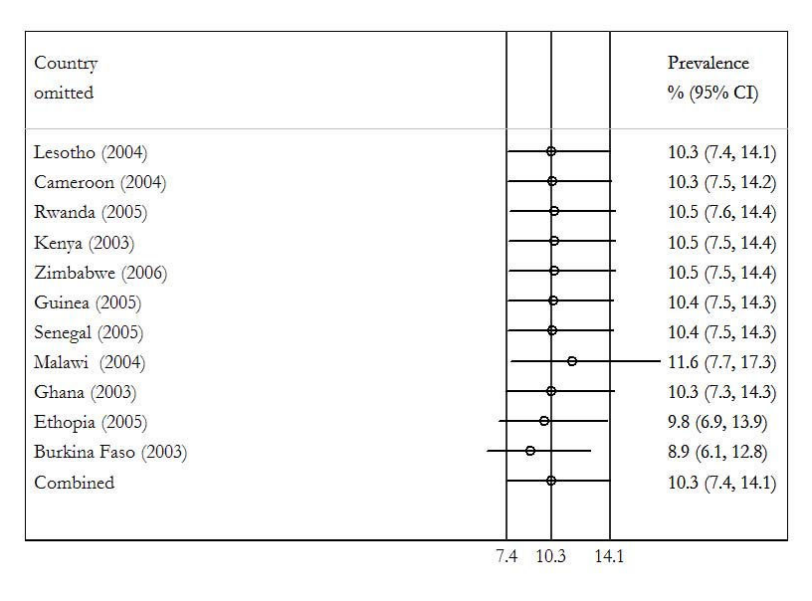
Plot indicating the influence of each country on the overall pooled result- "leave-one-country-out" sensitivity analysis.

To evaluate the stability of the meta-analysis, leave-one-"country"-out sensitivity analysis was performed (Figure 2). In this analysis, the overall prevalence was calculated, removing one country at a time. This shows the studies conducted in Malawi, Ethiopia, and Burkina Faso had the greatest influence on combined pooled prevalence of HIV-related malnutrition. Omitting studies from Ethiopia and Burkina Faso reduces the pooled prevalence to 9.8% (95% CI; 6.9% to 13.9%) and 8.9% (95% CI; 6.1% to 12.8%) respectively. While omitting study from Malawi increased the pooled prevalence to 11.6% (95% CI; 7.5% to 17.3%). However, the confidence intervals do change materially with exclusion of any of these countries, which remains within the 95% confidence interval of the overall estimate for all countries (Figure 2). This analysis confirmed the stability of the results.

### Sub-group analyses

To gain further insight into the prevalence of HIV-associated malnutrition, the prevalence estimates were disaggregated into difference socioeconomic status as shown in Table 3.

**Table 3:** Sub-group analyses by selected socioeconomic characteristics

***Category***	***Unweighted prevalence % (95% CI)***	***Weighted pooled prevalence %(95% CI)***
**Education**		
No education	10.7 [6.1, 15.3]	17.5 [16.7, 18.4]
Primary	8.3 [5.5, 11.0]	10.2 [9.2, 11.2]
Secondary or higher	5.9 [4.0, 7.7]	7.2 [6.2, 8.5]
**Occupation**		
Not working	9.1 [5.7, 12.6]	11.6 [10.7, 12.5]
White collar	4.8 [2.3, 7.2]	9.0 [7.5, 10.8]
Manual/agricultural	8.3 [4.3, 12.3]	17.5 [16.6, 18.5]
**Residence**		
Urban	5.4 [3.7, 7.0]	6.8 [5.9, 7.9]
Rural	9.4 [5.4, 13.4]	16.3 [15.7, 17.1]
**Wealth index**		
Poorest	11.3 [6.3, 16.3]	18.1 [16.8, 19.3]
Poorer	9.8 [5.7, 13.9]	16.5 [15.2, 17.9]
Middle	8.7 [4.5, 12.9]	17.0 [16.5, 19.4]
Richer	7.5 [4.2, 10.9]	13.3 [11.9, 14.9]
Richest	4.9 [3.2, 6.7]	7.2 [6.1, 8.5]
**Overall**	7.7 [4.8, 10.6]	10.3 [7.4, 14.1]

As expected, the prevalence of HIV-associated malnutrition decreased monotonically as one move up the wealth index quintiles. Similarly, the pooled prevalence of HIV-related malnutrition decreased with increasing level of education. The prevalence of underweight was higher among HIV-positive women living in the rural areas compared to their urban counterparts (6.8% versus 16.3%). Professionally employed women were less likely to be underweight than skilled or unskilled manual workers (9.0% versus 17.5%) and women not employed (9.0% versus 11.6%).

## Discussion

### Main findings

This meta-analysis of DHS has brought together evidence from 11 countries in sub-Saharan Africa conducted between 2003 and 2006. These surveys have important limitation in term of number of HIV-positive women, thus inferences have been limited by a lack of precision. The study therefore aimed to improve the precision of the estimate of prevalence of HIV-related malnutrition by carrying out a meta-analysis. Pooling the prevalence estimates of 11 countries yielded an overall prevalence of 10.3% (95% CI 7.4% to 14.1%). This study found a strong pattern of decreasing level of proportion of HIV-positive women with low body mass index with increasing level of education. Even more evident is the pattern of decreasing proportion of low body weight among HIV-positive women with increasing wealth index. Type of resident also displays distinctive patterns. HIV-positive women living in rural areas were decidedly thinner than those living in the urban areas.

Greater levels of malnutrition are also evident among women not employed and manual unskilled professionals compared to women in professional field. These findings are consistent with data from other studies that have examined these socioeconomic factors in the general population [30-36].

### Strengths and limitations of the study

There are a number of caveats to be considered when interpreting these results. Some criticize the aggregation of different study findings, particularly those with different levels of methodological quality [23]. Caution is essential when interpreting Cochran Q statistic, the statistical power of tests for heterogeneity are, in most cases, very low due to the small number of combined studies [37,38], implying that heterogeneity may be present even if the Q statistics is not statistically significant [28,39,40]. One important limitation is that DHS surveys do not collect data on household income or expenditure, the traditional indicators used to measure wealth. The assets-based wealth index used here is only a proxy indicator for household economic status, and it does not always produce results similar to those obtained from direct measurements of income and expenditure where such data are available or can be collected reliably [21,22]. Another limitation of the present analysis relates to the use of body mass index (BMI) as the only measure of malnutrition. Due to lack of appropriate data on dietary intake and food or calorie consumption, low body mass index among HIV positive women was used as proxy for malnutrition. Cross-sectional data only allow looking at associations; it is impossible to assess directly how the relation between socioeconomic position and BMI may change over time [41].

Despite these limitations, the study strengths are significant. It is a large, population-based study with national coverage from 11 countries, and low heterogeneity among included studies suggesting appropriateness of pooling results from different countries. Meta-analysis has numerous strengths. It allows for synthesis of study findings and comparison of findings across multiple studies [42]. Meta-analysis allows researchers to arrive at conclusions that are more accurate and more credible than can be presented in any one primary study or in a non-quantative, narrative review [43]. Also, meta-analysis can be used to highlights gaps or limitations in the research literature. The DHS have some important advantages when compared with other surveys. They are often nationally representative, allowing for conclusions that cover the entire nation [44]. In addition, same variable is operationalized in the same way and making it possible for numerical values comparable across countries. Overall, the number of included studies and geographic and socioeconomic diversities constitute a good yardstick for the region and help strengthen the findings from the study. In addition, low heterogeneity among studies suggests that the result of the meta-analysis is consistent and allows for generalisability of the results to other countries in sub-Saharan Africa.

## Conclusion

This meta-analysis adds to our knowledge on HIV-related malnutrition among women in sub-Saharan Africa by providing evidence of variations in prevalence estimates by wealth status, education attainment, occupation, and type of residence (rural/urban). The pooled prevalence values provided in this study can be used as an estimate of baseline probability in an evidence-based approach, to help clinicians, researchers, and policy makers make informed decision about prevention and treatment of HIV-related malnutrition. The observed socioeconomic disparities in prevalence of HIV-related malnutrition can help provide more information about population subgroups in particular need and high risk groups, which may in turn lead to the development and implementation of more effective intervention programs. While these findings do not suggest a redirection of prevention programs from urban to rural areas, rich to poor people, educated women to less educated women, they do call for efforts to ensure that HIV-related malnutrition prevention messages get across to all strata of society. Good nutrition cannot cure HIV/AIDS, but it can bolster the immune system and therefore postpone the onset of the disease and help HIV-infected people lead longer, healthier, more productive lives.

## Competing interests

The authors declare that they have no competing interests.

## Authors' contributions

OAU conceived the study, extracted the data, did the meta-analysis and interpretation, and wrote the first and final draft of the manuscript.

## Pre-publication history

The pre-publication history for this paper can be accessed here:


